# Chondrodystophia Fetalis

**Published:** 1914-08

**Authors:** 


**Chondrodystophia Fetalis.** Young, in Archives of Pediatrics, May, 1914, describes two cases, one of the hyperplastic form and one of the hypoplastic form of this disease.—C. G. L-W.

